# Validation Analysis of the Polish-Translated Version of EmPHasis-10 Health-Related Quality of Life Questionnaire in Patients with Pulmonary Arterial Hypertension

**DOI:** 10.3390/jcm15052020

**Published:** 2026-03-06

**Authors:** Maria Wieteska-Miłek, Dominika Tkaczyk, Adam Torbicki, Joanna Orłowska, Marcin Kurzyna, Małgorzata Woźniak-Prus

**Affiliations:** 1 Centre of Postgraduate Medical Education, European Health Centre, Department of Pulmonary Circulation, Thromboembolic Diseases and Cardiology, ERN-Lung Member, ul. Borowa 14/18, 05-400 Otwock, Poland; adam.torbicki@ecz-otwock.pl (A.T.); joanna-orlowska@ecz-otwock.pl (J.O.); marcin.kurzyna@ecz-otwock.pl (M.K.); 2 Department of Internal Medicine, Hospital in New Mazovian Manor, ul. Miodowa 2, 05-100 Nowy Dwór Mazowiecki, Poland; 3 Faculty of Psychology, University of Warsaw, ul. Banacha 2D, 02-097 Warsaw, Poland; malgorzata.wozniak-prus@psych.uw.edu.pl

**Keywords:** pulmonary arterial hypertension, quality of life, prognostic parameters, emphasis-10

## Abstract

**Background/Objectives:** Pulmonary arterial hypertension (PAH) impacts various aspects of patients’ lives. Some questionnaires assessing health-related quality of life are specific to PAH patients. The aims of the study were to translate and investigate the factor structure and psychometric properties of the Polish version of the EmPHasis-10 health-related quality of life questionnaire in a group of adults with PAH. Construct validity was explored by the relationship with results of the 36-Item Short Form Survey (SF-36) and non-invasive prognostic factors: WHO functional class, 6 min walk distance (6MWD) and NTproBNP level were measured. **Methods:** In a single-center study, PAH patients were included. The diagnosis of PAH was confirmed by right heart catheterization. The demographic and clinical data were obtained. The EmPHasis-10 and the SF-36 questionnaires were administered to all patients. **Results:** Data from 120 PAH patients, median age 57 (IQR 45–68.7) years, 88 (73%) women, were obtained. Most of the patients suffered from IPAH (73, 61%). Results revealed a unidimensional structure of the EmPHasis-10 questionnaire and demonstrated satisfactory reliability (Cronbach α = 0.94). The EmPHasis-10 showed an adequate relationship with both SF-36 dimensions and three non-invasive prognostic parameters, i.e., WHO functional class, 6MWD and NTproBNP level. Regression analysis indicated that the 6MWD was the only predictor of the EmPHasis-10. **Conclusions:** The obtained results showed very good psychometric properties and adequate internal consistency of the Polish version of EmPHasis-10 in PAH patients. The results showed a unidimensional structure and very good psychometric properties, including satisfactory internal consistency and external validity of the Polish version of the EmPHasis-10 scale in patients with PAH.

## 1. Introduction

Pulmonary arterial hypertension (PAH) is a progressive chronic disease characterized by remodeling of the pulmonary arteries and progressive right heart failure, with a high mortality rate [1,2,3]. Pulmonary arterial hypertension affects various aspects of a patient’s quality of life (QoL). The main PAH symptoms are exertional dyspnea, fatigue, and weakness [4,5]. Disease progression, possible treatment side effects, patient immobility, social isolation, dependence on others, and increased risk of mental disturbance, anxiety and depression may have an impact on the mental and physical components of quality of life [6,7].

Patient-Reported Outcome Measures (PROMs) are questionnaires or surveys used to obtain information about a patient’s health status directly from the patient’s perspective [8]. The 36-Item Short Form Survey (SF-36) can be used to assess the quality of life in patients with pulmonary hypertension (PH) and other chronic diseases [9,10,11]. Current pulmonary hypertension guidelines recommend measuring and incorporating PROMs into the diagnostic and treatment processes of PAH patients. Specific tools validated in the PAH group of patients are recommended [2,12,13] and they include: the Cambridge Pulmonary Hypertension Outcome Review (CAMPHOR) [14], Pulmonary Arterial Hypertension-Symptoms and Impact (PAH-SYMPACT) [15], the EmPHasis-10 [16] and, Living with PH [17]. It is evident that these disease-specific PROMs reflect QoL, functional status, clinical deterioration, and prognosis in PAH [8,16,18,19]. The EmPHasis-10 questionnaire was selected for validation in Polish PAH patients due to its brevity and simplicity. Preliminary assessments in patients familiar with SF-36, PAH-SYMPACT, and CAMPHOR indicated that EmPHasis-10 was the most user-friendly and appeared to be the most practical for routine clinical use.

The aim of the present study was to explore the psychometric properties of the EmPHasis-10 scale in the Polish patient population. The factor structure and reliability of the Polish version of the EmPHasis-10 scale were examined. Moreover, construct validity was assessed based on the relationship with non-invasive prognostic factors, which form part of the COMPERA-2 four-strata risk score in PAH patients and with the SF-36, a tool measuring QoL not specific to PAH, all of which were completed at the same time as the EmPhasis-10 questionnaire.

## 2. Materials and Methods

### 2.1. Study Design

The native Polish-speaking patients with PAH confirmed according to the current European Society of Cardiology Guidelines 2022, aged ≥18 years, with no change in World Health Organization (WHO) functional class or change in PH-specific treatment for the past 3 months prior to recruitment, were included in the study. 

Demographic and clinical data were collected from the medical history. All participants gave informed written consent to participate in the study. The study protocol was approved by the Bioethics Committee of the Center of Postgraduate Medical Education in accordance with the Declaration of Helsinki (number KBE 104/2024, date of approval: 11 December 2024). Eligible patients were invited to complete the two questionnaires: the EmPHasis-10 and the 36-item Short Form (SF-3) generic QoL measurement. 

The EmPHasis-10 is a psychometric tool that consists of 10 items dedicated to PH patients. Answers were given on a scale ranging from 0 to 5. Each patient chose a point from 0 to 5 for each statement and could get a total score of 0 to 40 points. There is a direct correlation between the points accumulated and the Quality of Life; that is to say that the greater the points, the poorer the QoL [16].

Permission from the Pulmonary Hypertension Association in the UK to check the Polish-translated version of the EmPHasis-10 was obtained before the study. The Polish version of the EmPHasis-10 is included in the Supplementary Material, Figure S1. The procedure for the validation analysis of the Polish version of the EmPHasis-10 health questionnaire started with checking the translation (translating the questionnaire forward, translating the questionnaire backward), which was then followed by the psychometric validation. The original version of the EmPHasis-10 questionnaire was obtained from the Pulmonary Hypertension Association in the United Kingdom and was then translated by a bilingual translator. EmPHasis-10 was translated into Polish and culturally adapted in accordance with established guidelines for cross-cultural research instrument translation [20]. The adaptation process included a forward translation followed by an independent blinded back-translation. Discrepancies between the original and back-translated versions were reviewed and resolved to ensure semantic and conceptual equivalence. The EmPHasis-10 questionnaire uses very simple and concise language. It was completed by patients in the presence of a nurse. None of the patients reported any difficulties or uncertainties while completing the questionnaire.

The SF-36 consists of 8 scales: physical functioning (PF), role-physical (RP), bodily pain (BP), general health (GH), vitality (VT), social functioning (SF), role-emotional (RE) and mental health (MH). The first four summarized components were used for evaluating the physical component of the SF-36 (PCS): PCS = PF + RP + BP + GH. The following four components, summarized, were used to evaluate the mental component of SF-36 (MCS): MCS = VT + SF + RE + MH. The higher the points, the better the QoL [9,11]. The license number QM 057109 was obtained for using the 36-item Short Form (SF-36,v.2). Three non-invasive parameters: World Health Organization Functional Class (WHO-FC), six-minute walk distance (6MWD), and level of N-terminal pro-brain natriuretic peptide (NT-proBNP) were used to calculate the risk of one year of death due to PH based on the COMPERA-2 model [1,21]. All non-invasive parameters were assessed at baseline at the same time as QoL.

### 2.2. Statistical Analysis

All statistical analyses were performed using IBM SPSS Statistics (version 30), Armonk, NY, USA. Normality of data distribution was assessed using histograms, the Shapiro–Wilk test and analyses of kurtosis and skewness. Since only two variables—PCS (physical component of the SF-36) and the 6MWD had a distribution close to normal, the non-parametric correlation tests were used. The factor structure of the EmPHasis-10 scale was investigated by performing an Exploratory Factor Analysis (EFA) using the Principal Component Analysis to determine the number of factors. The sample size for EFA is acceptable, as it falls within the recommended 20:1 ratio of cases to variables [22,23]. The scree plot, as well as Cattell and Kaiser criteria, were also implemented to extract the number of components [24,25]. The Kaiser–Meyer–Olkin Measure of Sampling Adequacy (KMO = 0.929) and Bartlett’s Test of Sphericity (χ^2^(45) = 857.995; *p* < 0.001) confirmed the appropriateness for conducting the EFA [26]. The internal consistency of the scale was explored using such coefficients of reliability as Cronbach’s alpha [27]. It is widely accepted that a coefficient over 0.70 is considered satisfactory [28]. Correlation coefficients were calculated to evaluate construct validity in terms of the relationship between the outcome of the Polish version of the EmPHasis-10 scale and other clinical equivalent variables. Correlation coefficients between 0 and 0.2 were considered as very weak, 0.2–0.39 as weak, 0.4–0.59 as moderate, 0.6–0.79 as strong, and 0.80–1.0 as very strong.

## 3. Results

### 3.1. Patient Characteristics

One hundred and twenty PAH patients, with a median (IQR) age of 57.4 (45–69) years, were included in the study. The majority (73.3%, *n* = 88) of participants were females. Most of them (61%) suffered from IPAH. The median duration of PAH was 4.3 (1.7–8.9) years. No concomitant disease was reported in 21 patients (17.5%). The remaining 82.5% of PAH patients had at least one concomitant disease or cardiovascular risk factor. Among 73 patients with IPAH, 18 were initially responders in the vasoreactivity test and received diltiazem as first-line therapy. Of this subgroup, only two were long-term responders and received diltiazem alone at the time of completing the EmPHasis-10, while 16 patients required the addition of targeted therapy to diltiazem. Baseline patient characteristics are presented in Table 1.

### 3.2. The Descriptive Statistics and Correlation Between Items

The descriptive statistics and correlation between the EmPHasis-10 items are presented in Table 2. All correlations were significant (*p* < 0.01). The correlation analysis results showed positive intercorrelations between all items of the EmPHasis-10 scale.

### 3.3. EmPHasis-10 Factor Structure and Reliability

An exploratory factor analysis with an oblique (Oblimin) rotation was conducted because the factors were expected to be correlated. Upon examining the scree plot and eigenvalues (greater than 1.0), the initial analysis indicated a one-factor solution. The unidimensional structure explained 65.2% of the total variance. The internal consistency of the scale, as measured by Cronbach’s alpha in the current sample, was 0.94. Table 3 presents the factor loadings from the Exploratory Factor Analysis. All loadings are higher than 0.7, which indicates that all items load significantly on the factor and that none of them should be removed. Conversely, all items refer to dimensions of functioning that are important for the quality of life of patients diagnosed with PAH.

### 3.4. Construct Validity of the EmPHasis-10

Firstly, the relationships between the Polish version of the EmPHasis-10 and other measures of the quality of life (SF-36) were examined (Table 4). The obtained results revealed that the EmPHasis-10 exhibits a significant, moderate and negative correlation with both dimensions of the SF-36—physical (PCS) and mental (MCS). The strength of the correlation is similar. This indicates that lower EmPHasis-10 scores are associated with a better perception of the physical and mental dimensions of quality of life.

Secondly, the links between the EmPHasis-10 scores and sample characteristics such as age, gender and disease duration were explored. Only one significant correlation was found—patients’ age was positively and weakly correlated (*ρ* = 0.32, *p* < 0.01). Thirdly, the relationships between the EmPHasis-10 scores and non-invasive prognostic parameters were examined (Table 5). The obtained results indicated a moderate positive correlation between the EmPHasis-10 and the WHO functional class (moderate strength) and a very weak correlation with NTproBNP. The 6MWD parameter showed a negative, moderate correlation with the EmPHasis-10. The correlation between COMPERA-2 and the EmPHasis-10 was positive and moderate. The pattern of the relationship between the SF-36 dimensions and non-invasive prognostic parameters was the opposite of that for the EmPHasis-10. The SF-36 Physical Component was strongly correlated with the WHO functional class and the 6MWD, moderately correlated with COMPERA-2 and weakly correlated with NTproBNP. The SF-36 Mental Component was moderately correlated only with the WHO functional class, and the 6MWD was moderately correlated with COMPERA-2.

### 3.5. Regression Analysis of Best Predictors in EmPHasis-10

**Figure 1:**
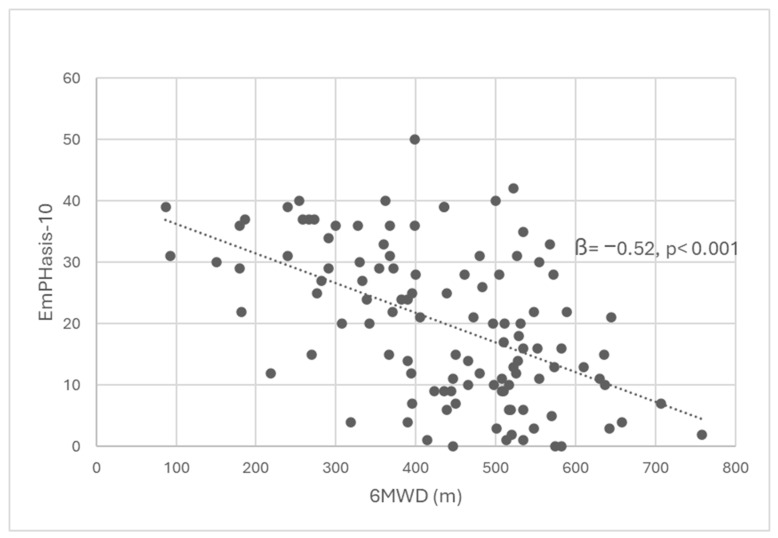
Relationship between six-minute walk distance (6MWD) and quality of life measured by EmPHasis-10 questionnaire. 6MWD: 6 min walk distance.

In order to find the best predictors of the EmPHasis-10 among non-invasive prognostic parameters and demographic variables (age, gender, disease duration), stepwise regression analysis was performed. The results indicated that only one model was significant, with the 6MWD parameter emerging as the sole predictor of EmPHasis-10 outcomes, with beta = −0.52, *p* < 0.001 (Figure 1). Non-invasive prognostic parameters and demographic variables were excluded from the model by an automatic procedure based on the statistical significance parameters of these potential prognostic factors (Table 6). The direction of this relationship indicates that the higher the 6MWD parameter, the lower the EmPHasis-10 scores. This model demonstrated a good fit to the data (F (111.1) = 41.33, *p* < 0.001) and explained 26.5% of the EmPHasis-10 variance.

## 4. Discussion

This is the first study on the validation of the Polish version of the EmPHasis-10 scale dedicated to pulmonary hypertension patients in the Polish population. The internal consistency of the scale analysis showed satisfactory Cronbach’s α values for the entire Polish questionnaire: 0.94—similar to the original study introducing EmPHasis-10 [16]. The study confirmed that this tool is an adequate and psychometrically satisfactory one, with good validity and reliability in Polish PAH patients. In clinical practice, the use of PROMs dedicated to pulmonary hypertension is strongly recommended [12,13]. There are different questionnaires used by PH patients. Nonspecific PROM but widely used in PAH and CTEPH is the 36-item Short Form (SF-36) [9]. Specific PROMs include the EmPHasis-10 [16], Living with Pulmonary Hypertension (LPH) [17], PHASIS [29], Cambridge Pulmonary Hypertension Outcome Review (CAMPHOR) [14], Pulmonary Arterial Hypertension Symptoms and Impact (PAH-SYMPACT^®^)32 PRO questionnaire [15]. The last two have even been validated in Polish [30,31]. The EmPHasis-10 scale, compared to the CAMPHOR and PAH-SYMPACT^®^)32 PRO questionnaires are much simpler, shorter and seem to be the easiest to use in everyday medical practice [13,32]. The EmPHasis-10 has been translated from English into various languages, including Dutch, Spanish, French, German, Italian, and validated in Turkish, Japanese, and Chinese for patients suffering from PAH [33,34,35,36]. The Polish EmPHasis-10 has been found to demonstrate adequate reliability and validity when used to assess disease-specific QoL in Polish patients diagnosed with PAH.

Exploratory Factor Analysis of the Polish EmPHasis-10 revealed a unidimensional structure which is consistent with the original version [16]. All factor loadings were higher than 0.7, but differed in value. This means that all items significantly loaded on the main factor, and none of them were outliers, so they should not be removed. The study found that the EmPHasis-10 demonstrated high internal consistency, which is not surprising in the case of a single-factor tool with a short list of interrelated items. This indicates the consistency of the tool and the good selection of individual items specifically describing the dimensions of quality of life in patients with PAH. Additionally, an analysis of the content of the items does not suggest that they are redundant in describing the condition of PAH patients. Rawling et al. suggested that the EmPHasis-10 consists of three underlying latent variables, which are based on the loading of items: “fatigue” (Items 3, 4, and 5), “independence” (Items 7, 8, 9, and 10), and “breathlessness” (Items 1, 2, and 6) [37]. Contrary to the results obtained by Rawling et al., the EFA indicates a single-factor structure of the EmPhasis-10 in the Polish population without such a division.

The EmPHasis-10 exhibits a significant, moderate correlation with both dimensions of the SF-36 survey, physical (PCS; r = −0.57; *p* < 0.01) and mental (MCS; r = −0.55, *p* < 0.01), PROM, widely used in PAH and CTEPH but nonspecific to these diseases. In another study, in Chinese patients with PAH and connective tissue disease, the EmPHasis-10 items were strongly associated with PCS (r = −0.85, *p*< 0.001) and MCS (r = −0.81, *p*< 0.001) of the SF-36 scale [35]. The differences in the correlation between SF-36 scores and Emphasis-10 in patients with PAH, beyond cultural factors, may be attributable to several additional considerations. SF-36 is not a disease-specific PROM. In the Chinese cohort, only patients with PAH associated with systemic connective tissue disease were included, with systemic lupus erythematosus accounting for 57% of cases [35]. In contrast, most patients in our cohort (61%) had idiopathic PAH, while only 22.5% had PAH associated with systemic connective tissue disease. Furthermore, disease severity was greater in our cohort, with 51% of patients classified as WHO functional class III or IV, compared with 24% in the Chinese cohort. Patients in our cohort were also older, and 82% had comorbidities that could negatively affect quality of life.

EmPHasis-10 health-related quality of life score predicts outcomes in patients with idiopathic and connective tissue disease-associated pulmonary arterial hypertension [18]. In our study, we did not examine the prognostic significance of this PROM in PAH patients, but we examined the correlation with non-invasive prognostic factors. The moderate correlations are demonstrated between the EmPHasis-10 questionnaire and the COMPERA-2 risk score domains, which predict one-year mortality in PAH patients. We observe a moderate correlation between the EmPHasis-10 questionnaire and the 6MWD, and it is in concordance with other observations at baseline [18,38], and during the 6-month and 12-month follow-up [38]. The present study observes a moderate correlation between the EmPHasis-10 questionnaire and the WHO functional class. Similarly, a moderate correlation between the EmPHasis-10 questionnaire and the WHO functional class was found by Lewis et al., but a weak correlation was reported by Hendrics et al. [18,38]. In a long-term observation, a moderate correlation was observed between the EmPHasis-10 and NYHA at 6 and 12 months of follow-up [38]. The present study finds no correlation between the EmPHasis-10 and NT-pro BNP levels, in accordance with the findings of previous researchers [38].

Interestingly, stepwise regression analysis in our study, including COMPERA-2 scale components (6MWD, WHO functional class, NTproBNP level) and demographic variables (age, gender and disease duration) revealed that 6MWD was the only predictor of the EmPHasis-10 scores (beta −0.52, *p* < 0.001). This result may suggest that this indicator corresponds to the difficulties in functioning or limitations experienced by patients in their daily lives. It is therefore worth analyzing this relationship in a longitudinal study.

In reality, patients suffering from PAH are rarely administered PROMs in clinical settings [39]. It is understood that the EmPHasis-10 is the only PROM with some clinical practice research in support of its use in routine clinical practice [40]. It is not entirely clear how often QoL should be assessed in stable patients with PAH and how to incorporate the result in daily routine practice. However, it has been shown to provide additional, valuable information about the patient and to improve doctor–patient communication and collaboration [13].

### Limitations of the Study

The study was subject to certain limitations. The study was of a single-center nature, and patients completed the survey only once. On the other hand, a study was conducted in a large population with a rare disease, whose treatment is therefore concentrated in one center. The relatively small sample size may have influenced the stability and generalizability of the factor analysis results, especially the ability to detect potentially more complex factor structures. However, the validation was conducted in a cohort of patients with a rare condition (PAH), rather than in the general population, which inherently limits sample size. Despite these constraints, the procedure is considered acceptable according to the literature. In this study, the scale was not reassessed, which prevented evaluation of the longitudinal stability of EmPHasis-10. Although follow-up data collection has begun for a subset of patients, the results are still being compiled and will be reported in a future analysis.

## 5. Conclusions

The EmPHasis-10 is a valuable tool with appropriate validity and reliability for assessing disease-specific QoL in the Polish patients diagnosed with PAH. The EmPHasis-10, due to its simplicity, can be widely used in routine clinical practice and in clinical research. Importantly, the use of a reliable tool that is available in different language versions allows for conducting international research and its application.

## Figures and Tables

**Table 1:** Baseline characteristics of PAH patients, *n* = 120.

	Variable	Result
Gender, Female/Male, n (%)		88/32 (73/27)
Age, years (IQR)		57.4 (45–68.7)
Type of PAH, n (%)	IPAH	73 (61)
	PAH-CTD	27 (22.5)
	PAH-CHD	11 (9.1)
	HPAH	6 (5)
	PAH porto-pulmonary	2 (1.6)
	DPAH	1 (0.8)
Duration of PAH disease, years (IQR)		4.3 (1.7–8.9)
PAH treatment, n (%)	monotherapy	24 (20)
	two drugs	45 (37.5)
	three drugs	45 (37.5)
	four drugs	1 (0.08)
	diltiazem	18 (15)
Concomitant disease, n (%)	Arterial hypertension	62 (50.2)
	Diabetes	23 (19.1)
	Coronary artery disease	11 (9.2)
	Obesity, BMI ≥ 30 kg/m^2^	31 (25.8)
	Hyperlipidaemia or pharmacologic lipid treatment	54 (45)
	COPD	13 (10.8)
	Other lung disease (asthma, ILD, other)	9 (7.5)
	Smoking cigarettes, past or current	30 (25)
	Neoplasm, past or current	19 (15.8)
	History of depression or antidepressants	15 (12.5)
WHO functional class, n (%)	1	6 (5)
	2	53 (44.2)
	3	58 (48.3)
	4	3 (2.5)
Laboratory test	NTproBNP, pg/mL, median (IQR)	251 (94.4–732.5)
Six-minute walk test (*n* = 114)	6MWD, m; mean (SD)	436.8 (132.1)

Data are presented as n, mean (SD), or median (1st–3rd quartile). PAH: pulmonary arterial hypertension; DPAH—Drug- or toxin-induced pulmonary arterial hypertension; CTD: connective tissue disease; IPAH: idiopathic PAH; HPAH: heritable PAH; CTD-PAH: CTD-associated PAH; WHO: World Health Organization; 6MWD: 6 min walk distance; NT-proBNP: N-terminal pro-brain natriuretic peptide; COPD—Chronic obstructive pulmonary disease, BMI—body mass index.

**Table 2:** Descriptive statistics and correlations between items of the EmPHasis-10.

Items	M	SD	[1]	[2]	[3]	[4]	[5]	[6]	[7]	[8]	[9]	[10]
[1] Emph-1	2.12	1.66	1									
[2] Emph-2	1.61	1.69	0.69	1								
[3] Emph-3	3	1.68	0.59	0.57	1							
[4] Emph-4	2.46	1.47	0.66	0.64	0.82	1						
[5] Emph-5	2.62	1.38	0.47	0.48	0.7	0.7	1					
[6] Emph-6	2.11	1.81	0.61	0.59	0.57	0.61	0.5	1				
[7] Emph-7	1.7	1.73	0.6	0.49	0.53	0.57	0.53	0.59	1			
[8] Emph-8	2.32	1.52	0.65	0.53	0.64	0.67	0.59	0.55	0.6	1		
[9] Emph-9	1.84	1.68	0.63	0.62	0.66	0.69	0.58	0.67	0.63	0.77	1	
[10] Emph-10	1.4	1.51	0.6	0.55	0.58	0.64	0.55	0.61	0.67	0.64	0.73	1

Emph—item from EmPHasis-10 scale; M—mean; SD—standard deviation.

**Table 3:** Exploratory factor analysis (EFA) factor loadings (Cronbach’s alpha) of the EmPHasis-10 items.

Item	Factor Loadings
Emphasis 1	0.774
Emphasis 2	0.724
Emphasis 3	0.811
Emphasis 4	0.860
Emphasis 5	0.726
Emphasis 6	0.743
Emphasis 7	0.728
Emphasis 8	0.813
Emphasis 9	0.857
Emphasis 10	0.787

**Table 4:** Correlations between quality of life questionnaires the EmPHasis-10 and SF-36.

	EmPHasis-10	SF-36 PCS	SF-36 MCS
EmPHasis-10	1		
SF-36 PCS	−0.57 **	1	
SF-36 MCS	−0.55 **	0.35 **	1

** *p* < 0.01; SF-36, 36-item Medical Outcomes Study Short Form Survey; MCS, Mental Component Summary; PCS, Physical Component Summary.

**Table 5:** Correlations between the EmPHasis-10 and SF-36 questionnaires and non-invasive prognostic parameters in the study sample.

	WHO Functional Class	6MWD (m)	NTproBNP (pg/mL)	COMPERA-2 Score v.2
EmPHasis-10	0.46 **	−0.51 **	0.19 *	0.47 **
SF-36 PCS	−0.64 **	0.64 **	−0.19 *	−0.59 **
SF-36 MCS	−0.35 **	0.39 **	−0.08	−0.36 **

* *p* < 0.05, ** *p* < 0.01; SF-36, 36-item Medical Outcomes Study Short Form Survey; MCS, Mental Component Summary; PCS, Physical Component Summary; WHO: World Health Organization functional class; 6MWD: 6 min walk distance; NT-proBNP: N-terminal pro-brain natriuretic peptide; COMPERA-2 score.

**Table 6:** Regression analysis of noninvasive predictors of the EmPHasis-10—variables excluded from the model.

Variable	*ß*	*p*	r
Age	−0.21	0.06	−0.18
Gender	0.11	0.19	0.13
Duration of PAH disease	−0.04	0.65	−0.04
WHO functional class	0.07	0.38	0.08
NTproBNP (pg/mL)	0.06	0.61	0.05

Beta (ß), *p*-values and partial correlation coefficient for variables excluded from model; NT-proBNP: N-terminal pro-brain natriuretic peptide; WHO: World Health Organization.

## Data Availability

The original contributions presented in this study are included in the article. Further inquiries can be directed to the corresponding author.

## Supplementary Materials

The following supporting information can be downloaded at: https://www.mdpi.com/article/10.3390/jcm15052020/s1, Figure S1: Polish version of the Emphasis-10 questionnaire.

## Abbreviations

The following abbreviations are used in this manuscript:

6MWD	6 min walk distance
PAH	pulmonary arterial hypertension
PH	pulmonary hypertension
PRO	patient-reported outcome
PROM	patient-reported outcome measures
QoL	quality of life
NTproBNP	N-terminal pro-B-type natriuretic peptide
SF-36	36-Item Short Form Survey
