# Supplementary material for: Validation Analysis of the Polish-Translated Version of EmPHasis-10 Health-Related Quality of Life Questionnaire in Patients with Pulmonary Arterial Hypertension
Source: J Clin Med. 2026 Mar 6;15(5):2020. doi: 10.3390/jcm15052020 (PMC12986267; doi:10.3390/jcm15052020)
Supplement: Supplementary file 1 [file jcm-15-02020-s001.zip › jcm-4127898-supplementary.pdf]

**Supplementary Material**

emPHasis10

Nr ident. (PESEL):

Imię i nazwisko:

Data urodzenia:

Ten kwestionariusz służy do ustalenia sposobu, w jaki nadciśnienie płucne wpływa na Pana/Pani życie. Proszę odpowiedzieć na każde pytanie, stawiając haczyk nad JEDNĄ LICZBĄ, która najlepiej opisuje Pani/Pana dotychczasowe doświadczenia związane z nadciśnieniem płucnym.

Przy każdym z poniższych stwierdzeń proszę zaznaczyć (✓) pole, które najlepiej opisuje Pana/Pani doświadczenia.

| | | | | | | | |
|---|---|---|---|---|---|---|---|
| Nie czuję frustracji z powodu występujących u mnie duszności. | 0 | 1 | 2 | 3 | 4 | 5 | Czuję dużą frustrację z powodu występujących u mnie duszności. |
| Duszności nigdy nie przerywają mi prowadzonych rozmów. | 0 | 1 | 2 | 3 | 4 | 5 | Duszności zawsze przerywają mi prowadzone rozmowy. |
| Nie muszę odpoczywać w ciągu dnia. | 0 | 1 | 2 | 3 | 4 | 5 | Zawsze muszę odpoczywać w ciągu dnia. |
| Nie czuję się wyczerpany(-a). | 0 | 1 | 2 | 3 | 4 | 5 | Zawsze czuję się wyczerpany(-a). |
| Mam dużo energii. | 0 | 1 | 2 | 3 | 4 | 5 | Nie mam w ogóle energii. |
| Gdy wchodzę po schodach na pierwsze piętro, nie mam duszności. | 0 | 1 | 2 | 3 | 4 | 5 | Gdy wchodzę po schodach na pierwsze piętro, mam silne duszności. |
| Czuję się pewnie w miejscach publicznych/tłumie, pomimo występującego u mnie nadciśnienia płucnego. | 0 | 1 | 2 | 3 | 4 | 5 | W ogóle nie czuję się pewnie w miejscach publicznych/tłumie z powodu występującego u mnie nadciśnienia płucnego. |
| Nadciśnienie płucne nie ogranicza mojego życia. | 0 | 1 | 2 | 3 | 4 | 5 | Nadciśnienie płucne całkowicie ogranicza moje życie. |
| Jestem niezależny(-a). | 0 | 1 | 2 | 3 | 4 | 5 | Jestem całkowicie zależny(-a). |
| Nigdy nie czuję, że jestem ciężarem. | 0 | 1 | 2 | 3 | 4 | 5 | Zawsze czuję, że jestem ciężarem. |

Łącznie:

Data:

Copyright © 2013 PHA UK. Data publikacji październik 2013 r., wersja 2.0

EmPHasis-10 AssessmentTool (Poland-Polish) 07JUL2021 FINAL

Figure S1: Polish version of the Emphasis-10 questionnaire.
